# Photosynthetic Physiology of Blue, Green, and Red Light: Light Intensity Effects and Underlying Mechanisms

**DOI:** 10.3389/fpls.2021.619987

**Published:** 2021-03-05

**Authors:** Jun Liu, Marc W. van Iersel

**Affiliations:** Horticultural Physiology Laboratory, Department of Horticulture, University of Georgia, Athens, GA, United States

**Keywords:** photosynthesis, quantum yield of CO_2_ assimilation, light spectrum, photosynthetic photon flux density, electron transport, *V*_c,max_, light intensity, light quality

## Abstract

Red and blue light are traditionally believed to have a higher quantum yield of CO_2_ assimilation (*QY*, moles of CO_2_ assimilated per mole of photons) than green light, because green light is absorbed less efficiently. However, because of its lower absorptance, green light can penetrate deeper and excite chlorophyll deeper in leaves. We hypothesized that, at high photosynthetic photon flux density (*PPFD*), green light may achieve higher *QY* and net CO_2_ assimilation rate (*A*_n_) than red or blue light, because of its more uniform absorption throughtout leaves. To test the interactive effects of *PPFD* and light spectrum on photosynthesis, we measured leaf *A*_n_ of “Green Tower” lettuce (*Lactuca sativa*) under red, blue, and green light, and combinations of those at *PPFD*s from 30 to 1,300 μmol⋅m^–2^⋅s^–1^. The electron transport rates (*J*) and the maximum Rubisco carboxylation rate (*V*_c,max_) at low (200 μmol⋅m^–2^⋅s^–1^) and high *PPFD* (1,000 μmol⋅m^–2^⋅s^–1^) were estimated from photosynthetic CO_2_ response curves. Both *QY*_m,inc_ (maximum *QY* on incident *PPFD* basis) and *J* at low *PPFD* were higher under red light than under blue and green light. Factoring in light absorption, *QY*_m,abs_ (the maximum *QY* on absorbed *PPFD* basis) under green and red light were both higher than under blue light, indicating that the low *QY*_m,inc_ under green light was due to lower absorptance, while absorbed blue photons were used inherently least efficiently. At high *PPFD*, the *QY*_inc_ [gross CO_2_ assimilation (*A*_g_)/incident *PPFD*] and *J* under red and green light were similar, and higher than under blue light, confirming our hypothesis. *V*_c,max_ may not limit photosynthesis at a *PPFD* of 200 μmol m^–2^ s^–1^ and was largely unaffected by light spectrum at 1,000 μmol⋅m^–2^⋅s^–1^. *A*_g_ and *J* under different spectra were positively correlated, suggesting that the interactive effect between light spectrum and *PPFD* on photosynthesis was due to effects on *J*. No interaction between the three colors of light was detected. In summary, at low *PPFD*, green light had the lowest photosynthetic efficiency because of its low absorptance. Contrary, at high *PPFD*, *QY*_inc_ under green light was among the highest, likely resulting from more uniform distribution of green light in leaves.

## Introduction

**FIGURE 1:**
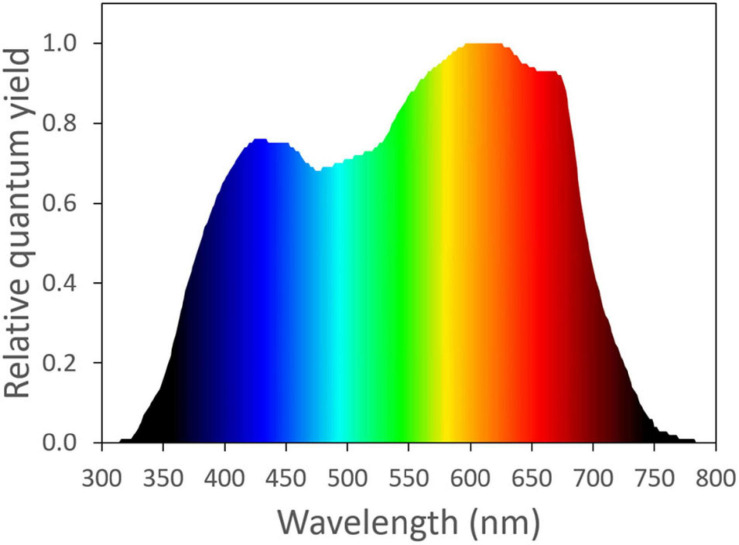
The normalized action spectrum of the maximum quantum yield of CO_2_ assimilation for narrow wavebands of light from ultra-violet to far-red wavelengths (McCree, 1971). Redrawn using data from Sager et al. (1988).

The photosynthetic activity of light is wavelength dependent. Based on McCree’s work (McCree, 1971, 1972), photosynthetically active radiation is typically defined as light with a wavelength range from 400 to 700 nm. Light with a wavelength shorter than 400 nm or longer than 700 nm was considered as unimportant for photosynthesis, due to its low quantum yield of CO_2_ assimilation, when applied as a single waveband (Figure 1). Within the 400–700 nm range, McCree (1971) showed that light in the red region (600–700 nm) resulted in the highest quantum yield of CO_2_ assimilation of plants. Light in the green region (500–600 nm) generally resulted in a slightly higher quantum yield than light in the blue region (400–500 nm) (Figure 1; McCree, 1971). The low absorptance of green light is partly responsible for its low quantum yield of CO_2_ assimilation. Within the visible spectrum, green leaves have the highest absorptance in the blue region, followed by red. Green light is least absorbed by green leaves, which gives leaves their green appearance (McCree, 1971; Zhen et al., 2019).

Since red and blue light are absorbed more strongly by photosynthetic pigments than green light, they are predominantly absorbed by the top few cell layers, while green light can penetrate deeper into leaf tissues (Nishio, 2000; Vogelmann and Evans, 2002; Terashima et al., 2009; Brodersen and Vogelmann, 2010), thus giving it the potential to excite photosystems in deeper cell layers. Leaf photosynthesis may benefit from the more uniform light distribution throughout a leaf under green light. Absorption of photons by chloroplasts near the adaxial surface may induce heat dissipation of excess excitation energy in those chloroplasts, while chloroplasts deeper into the leaf receive little excitation energy (Sun et al., 1998; Nishio, 2000). Blue and red photons, therefore, may be used less efficiently and are more likely to be dissipated as heat than green photons.

The misconception that red and blue light are used more efficiently by plants than green light still occasionally appears (Singh et al., 2015), often citing McCree’s action spectrum or the poor absorption of green light by chlorophyll extracts. The limitations of McCree’s action spectrum were explained in his original paper: the quantum yield was measured under low photosynthetic photon flux density (*PPFD*), using narrow waveband light, and expressed on an incident light basis (McCree, 1971), but these limitations are sometimes ignored. The importance of green light for photosynthesis has been well established in more recent studies (Sun et al., 1998; Nishio, 2000; Terashima et al., 2009; Hogewoning et al., 2012; Smith et al., 2017).

From those studies, one trend has emerged that has not received much attention: there is an interactive effect of light quality and intensity on photosynthesis (Sun et al., 1998; Evans and Vogelmann, 2003; Terashima et al., 2009). At low *PPFD*, green light has the lowest *QY*_inc_ (quantum yield of CO_2_ assimilation on incident light basis) because of its low absorptance; at high *PPFD*, on the other hand, red and blue light have a lower *QY*_inc_ than green light, because of their high absorptance by photosynthetic pigments, which shifts much of the light absorption closer to the upper leaf surface. This reduces both the quantum yield of CO_2_ assimilation in cells in the upper part of a leaf and light availability in the bottom part of a leaf.

The interactive effect between light quality and intensity was illustrated in an elegant study that quantified the differential quantum yield, or the increase in leaf CO_2_ assimilation per unit of additional light (Terashima et al., 2009). The differential quantum yield was measured by adding red or green light to a background illumination of white light of different intensities. At low background white light levels, the differential quantum yield of red light was higher than that of green light, due to the low absorptance of green light. But as the background light level increased, the differential quantum yield of green light decreased more slowly than that of red light, and was eventually higher than that of red light (Terashima et al., 2009). The red light was absorbed efficiently by the chloroplasts in the upper part of leaves. With a high background level of white light, those chloroplasts already received a large amount of excitation energy from white light and up-regulated non-photochemical quenching (NPQ) to dissipate excess excitation energy as heat, causing the additional red light to be used inefficiently. Green light, on the other hand, was able to reach the chloroplasts deeper in the mesophyll and excited those chloroplasts that received relatively little excitation energy from white light. Therefore, with high background white light intensity, additional green light increased leaf photosynthesis more efficiently than red light (Terashima et al., 2009).

In this paper, we present a comprehensive study to explore potential interactive effect of light intensity and light quality on C_3_ photosynthesis and underlying processes. We quantified the photosynthetic response of plants to blue, green, and red light over a wide *PPFD* range to better describe how light intensity and waveband interact. In addition, we examined potential interactions among blue, green, and red light, using light with different ratios and intensities of the three narrow waveband lights. To get a better understanding of the biochemical reasons for the effects of light spectrum and intensity on CO_2_ assimilation, we constructed assimilation – internal leaf CO_2_ (*C*_i_) response curves (*A/C_i_* curves) under blue, green, and red light, as well as combinations of the three narrow waveband lights at both high and low *PPFD*. We hypothesized that effects of different light spectra would be reflected in the electron transport rate (*J*) required to regenerate consumed ribulose 1,5-bisphosphate (RuBP), rather than the maximum carboxylation rate of ribulose-1,5-bisphosphate carboxylase/oxygenase (Rubisco) (*V*_c,max_).

## Materials and Methods

### Plant Material

Lettuce “Green Towers” plants were grown from seed in 1.7 L round pots filled with soilless substrate (Fafard 4P Mix, Sun Gro Horticulture, Agawam, MA, United States). The plants were grown in a growth chamber (E15, Conviron, Winnipeg, Manitoba, Canada) at 23.2 ± 0.8°C (mean ± SD), under white fluorescent light with a 14-hr photoperiod, vapor pressure deficit (VPD) of 1.20 ± 0.43 kPa and a *PPFD* of 200–230 μmol⋅m^–2^⋅s^–1^ at the floor level, and ambient CO_2_ concentration. Plants were sub-irrigated when necessary with a nutrient solution containing 100 mg⋅L^–1^ N, made with a complete, water-soluble fertilizer (Peter’s Excel 15-5-15 Cal-Mag fertilizer, Everris, Marysville, OH, United States).

### Leaf Absorptance, Transmittance, and Reflectance

Leaf absorptance was determined using a method similar to that of Zhen et al. (2019). Three plants were randomly selected. A newly expanded leaf from each plant was illuminated with a broad-spectrum halogen bulb (70W; Sylvania, Wilmington, MA, United States) for leaf transmittance measurement. Transmittance was measured with a spectroradiometer (SS-110, Apogee, Logan, UT, United States). The halogen light spectrum was taken as reference measurement with the spectroradiometer placed directly under the halogen bulb in a dark room. Then, a lettuce leaf was placed between the halogen bulb and spectroradiometer, with its adaxial side facing the halogen bulb and transmitted light was measured. Leaf transmittance was then calculated on 1 nm resolution. Light reflectance of the leaves was measured using a spectrometer with a leaf clip (UniSpec, PP systems, Amesbury, MA, United States). Light absorptance was calculated as 1−*reflectance*−*transmittance*. We verified that this method results in similar absorptance spectra as the use of an integrating sphere. Absorptance of each of the nine light spectra used in this study were calculated from the overall leaf absorptance spectrum and the spectra of the red, green, and blue LEDs.

### Leaf Photosynthesis Measurements

All gas exchange measurements were made with a leaf gas exchange system (CIRAS-3, PP Systems). Light was provided by the LEDs built into the chlorophyll fluorescence module (CFM-3, PP Systems). This module has dimmable LED arrays of different colors, with peaks at 653 nm [red, full width at half maximum (FWHM) of 17 nm], 523 nm (green, FWHM of 36 nm), and 446 nm (blue, FWHM of 16 nm). Nine different combinations of red, green, and blue light were used in this study (Table 1). Throughout the measurements, the environmental conditions inside the cuvette were controlled by the leaf gas exchange system. Leaf temperature was 23.0 ± 0.1°C, CO_2_ concentration was 400.5 ± 4.1 μmol⋅mol^–1^, and the VPD of air in the leaf cuvette was 1.8 ± 0.3 kPa (mean ± SD).

**TABLE 1:** List of light spectrum abbreviations and their spectral composition.

Light spectrum	Fraction of total photon flux (%)		
	Blue	Green	Red
100B	100	0	0
80B20G	80	20	0
20B80G	20	80	0
100G	0	100	0
80G20R	0	80	20
20G80R	0	20	80
100R	0	0	100
20B80R	20	0	80
16B20G64R	16	20	64

#### Photosynthesis – Light Response Curves

To explore photosynthetic efficiency of light with different spectra, we constructed light response curves for lettuce plants using each light spectrum. Lettuce plants were exposed to 10 *PPFD* levels ranging from 30 to 1,300 μmol⋅m^–2^⋅s^–1^ (30, 60, 90, 120, 200, 350, 500, 700, 1,000, and 1,300 μmol⋅m^–2^⋅s^–1^) in ascending orders for light response curves. Photosynthetic measurements were taken on 40–66 days old lettuce plants. Lettuce plants were taken out of the growth chamber and dark-adapted for 30 min. Starting from the lowest *PPFD*, one newly expanded leaf was exposed to all nine spectra. Net CO_2_ assimilation rate (*A*_n_) of the leaf was measured using the leaf gas exchange system. Under each light spectrum, three *A*_n_ readings were recorded at 10 s intervals after readings were stable (about 4–20 min depending on *PPFD* after changing *PPFD* and spectrum). The three *A*_n_ readings were averaged for analysis. After *A*_n_ measurements under all nine light spectra were taken, the leaf was exposed to the next *PPFD* level and *A*_n_ measurements were taken with the light spectra in the same order, until measurements were completed at all *PPFD* levels. Throughout the light response curves, *C*_i_ decreased with increasing *PPFD*, from 396 ± 10 μmol⋅mol^–1^ at a *PPFD* of 30 μmol⋅m^–2^⋅s^–1^ to 242 ± 44 μmol⋅mol^–1^ at a *PPFD* of 1,300 μmol⋅m^–2^⋅s^–1^. To account for the potential effect of plants and the order of the spectra on assimilation rates, the order of the different spectra was re-randomized for each light response curve, using a Latin square design with plant and spectrum as the blocking factors. Data were collected on nine different plants.

Regression curves (exponential rise to maximum) were fitted to the data for each light spectrum and replication (plant):

(1) $$A_{n}=A_{g,m\,a\,x}\times \left(1-{\text{e}}^{-Q\,Y_{m,i\,n\,c}\,\frac{P\,P\,F\,D}{A_{g,m\,a\,x}}}\right)-R_{d}$$

where *R*_d_ is the dark respiration rate, *QY*_m,inc_ is the maximum quantum yield of CO_2_ assimilation (initial slope of light response curve, mol of CO_2_ fixed per mol of incident photons) and *A*_g,max_ is the light-saturated gross assimilation rate. The *A*_n,max_ is the light-saturated net assimilation rate and was calculated as *A*_*n*,*max*_=*A*_*g*,*max*_-*R*_*d*_. The maximum quantum yield of CO_2_ assimilation was also calculated on absorbed light basis as $Q\,Y_{m,a\,b\,s}=\frac{Q\,Y_{m,i\,n\,c}}{l\,i\,g\,h\,t\,a\,b\,s\,o\,r\,p\,t\,a\,n\,c\,e}$.

The instantaneous quantum yield of CO_2_ assimilation based on incident *PPFD* (*QY*_inc_) was calculated as $\frac{A_{g}}{P\,P\,F\,D}$ for each *PPFD* at which *A*_n_ was measured, where the gross CO_2_ assimilation rate (*A*_g_) was calculated as *A*_*g*_=*A*_*n*_+*R*_*d*_. To account for differences in absorptance among the different light spectra, the quantum yield of CO_2_ assimilation was also calculated based on absorbed light base, as $Q\,Y_{a\,b\,s}=\frac{A_{g}}{P\,P\,F\,D\times l\,i\,g\,h\,t\,a\,b\,s\,o\,r\,p\,t\,a\,n\,c\,e}$, where light absorptance is the absorptance of lettuce leaves for each specific light spectrum. The *differential QY*, the increase in assimilation rate per unit of additional incident *PPFD*, was calculated as the derivative of Eq. 1:

(2) $$D\,i\,f\,f\,e\,r\,e\,n\,t\,i\,a\,l\,Q\,Y=Q\,Y_{m,i\,n\,c}\times {\text{e}}^{-Q\,Y_{m,i\,n\,c}\,\frac{P\,P\,F\,D}{A_{g,m\,a\,x}}}$$

#### Photosynthesis – Internal CO_2_ Response (*A/C_i_*) Curves

To explore the underlying physiological mechanisms of assimilation responses to different light spectra, we constructed *A/C_i_* curves. Typically, *A/C_i_* curves are collected under saturating *PPFD*. We collected *A/C_i_* curves at two *PPFD*s (200 and 1,000 μmol⋅m^–2^⋅s^–1^) to explore interactive effects of light spectrum and *PPFD* on the assimilation rate. At a *PPFD* of 200 μmol⋅m^–2^⋅s^–1^, red light has the highest *A*_n_ and green light the lowest *A*_n_, while at *PPFD* of 1,000 μmol⋅m^–2^⋅s^–1^, red and green light resulted in the highest *A*_n_ and blue light in the lowest *A*_n_.

We used the rapid *A/C_i_* response (RACiR) technique that greatly accelerates the process of constructing *A/C_i_* curves (Stinziano et al., 2017). We used a Latin square design, similar to the light response curves. *A/C_i_* curves were measured under the same nine spectra used for the light response curves. Nine lettuce plants were used as replicates. For each *A/C_i_* curve, CO_2_ concentration in the leaf cuvette started from 0 μmol⋅mol^–1^, steadily ramping to 1,200 μmol⋅mol^–1^ over 6 min. A reference measurement was also taken at the beginning of each replication with an empty cuvette to correct for the reaction time of the leaf gas exchange system. Post-ramp data processing was used to calculate the real *A* and *C*_i_ with the spreadsheet provided by PP systems, which yielded the actual *A/C_i_* curves with *C*_i_ range of about 100–950 μmol mol^–1^. Throughout the data collection, leaf temperature was 24.4 ± 1.3°C and VPD in the cuvette was 1.4 ± 0.2 kPa.

Curve fitting for *A/C_i_* curves was done by minimizing the residual sum of squares, following the protocol developed by Sharkey et al. (2007). Among our nine replicates, four plants did not show clear Rubisco limitations at low *PPFD* and for those plants Rubisco limitation (*V_c,max_*) was not included in the model (Sharkey et al., 2007). We therefore report *V*_c,max_ values for high *PPFD* only. The *J* was determined for all light spectra at both *PPFD*s. We therefore report *V*_c,max_ was determined for all light spectra only at high *PPFD*. The quantum yield of electron transport [*QY(J)*] was calculated on both incident and absorbed *PPFD* basis as $Q\,Y\,{\left(J\right)}_{i\,n\,c}=\frac{J}{P\,P\,F\,D}$ and $Q\,Y\,{\left(J\right)}_{a\,b\,s}=\frac{Q\,Y\,{\left(J\right)}_{i\,n\,c}}{l\,i\,g\,h\,t\,a\,b\,s\,o\,r\,p\,t\,a\,n\,c\,e}$, respectively. We did not estimate triose phosphate utilization, because the *A/C_i_* curves often did not show a clear plateau.

### Data Analysis

The *QY*_m,inc_, *QY*_m,abs_, and *A*_g,max_ were analyzed with ANOVA to determine the effects of light spectrum using SAS (SAS University Edition; SAS Institute, Cary, NC, United States). *A*_n_, *QY*_inc_, and *QY*_abs_ at each *PPFD* level and *V*_c,max_ and *J* estimated from *A/C_i_* curves were similarly analyzed with ANOVA using SAS. *A*_n_ at different *PPFD* levels were analyzed with regression analysis to detect interactive effect of blue, green, and red light on leaf assimilation rates using the fractions of red, blue, and green light as explanatory variables (JMP Pro 15, SAS Institute).

## Results

### Leaf Absorptance

**FIGURE 2:**
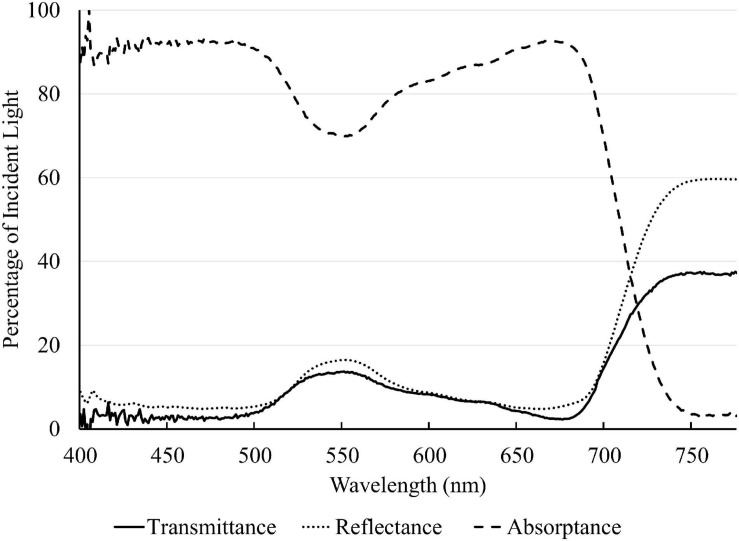
Light absorptance, reflectance, and transmittance spectrum of a newly fully expanded “Green Towers” lettuce leaf.

A representative spectrum of light absorptance, reflectance and transmittance of a newly fully expanded lettuce leaf is shown in Figure 2. In the blue region, 400–500 nm, the absorptance by “Green Towers” lettuce leaves was high and fairly constant, averaging 91.6%. The leaf absorptance decreased as the wavelength increased from 500 to 551 nm where the absorptance minimum was 69.8%. Absorptance increased again at longer wavelengths, with a second peak at 666 nm (92.6%). Above 675 nm, the absorptance decreased steadily to <5% at 747 nm (Figure 2). The absorptance spectrum of our lettuce leaves is similar to what McCree (1971) obtained for growth chamber-grown lettuce, with the exception of slightly higher absorptance in the green part of the spectrum in our lettuce plants. Using this spectrum, the absorptance of the blue, green, and red LED lights were calculated to be 93.2 ± 1.0%, 81.1 ± 1.9% and 91.6 ± 1.1%, respectively. Absorptance of all nine spectra was calculated based on their ratios of red, green, and blue light (Table 2).

**TABLE 2:** Light absorptance and transmittance of new fully expanded “Green towers” lettuce leaves under nine light spectra.

Light spectrum*	Light absorptance (%)	Light transmittance (%)
100B	93.2	2.2
80B20G	90.8	3.6
20B80G	83.6	7.8
100G	81.1	9.1
80G20R	83.2	8.1
20G80R	89.5	4.9
100R	91.6	3.9
20B80R	91.9	3.5
16B20G64R	89.8	4.7

See Figure 2 for the leaf absorptance spectrum. *See spectral composition in Table 1.

### Light Quality and Intensity Effects on Photosynthetic Parameters

**FIGURE 3:**
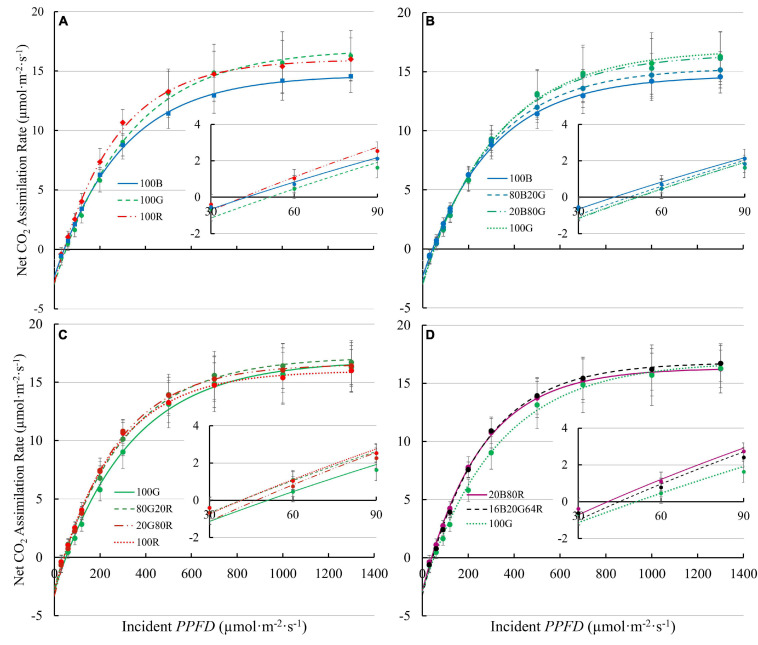
Net assimilation (*A*_n_) – light response curves of “Green Towers” lettuce under nine light spectra. Error bars represent the standard deviation (*n* = 9). Inserts show *A*_n_ against *PPFD* of 30-90 μmol⋅m^–2^⋅s^–1^s to better show the initial slopes of curves. The composition of the nine light spectra is shown in Table 1. The light spectra in the graphs are **(A)** 100B, 100G, and 100R; **(B)** 100B, 80B20G, 20B80G, and 100G; **(C)** 100G, 80G20R, 20G80R, and 100R; and **(D)** 20B80R, 16B20G64R, and 100G.

Light response curves of lettuce under all nine spectra are shown in Figure 3, with regression coefficients in Supplementary Table 1. It is worth noting that a few plants showed photoinhibition under 100B (decrease in *A*_n_ with *PPFD* > 1,000 μmol⋅m^–2^⋅s^–1^). Those data were excluded in curve fitting for light response curves to better estimate asymptotes. Photoinhibition was not observed under other spectra.

**FIGURE 4:**
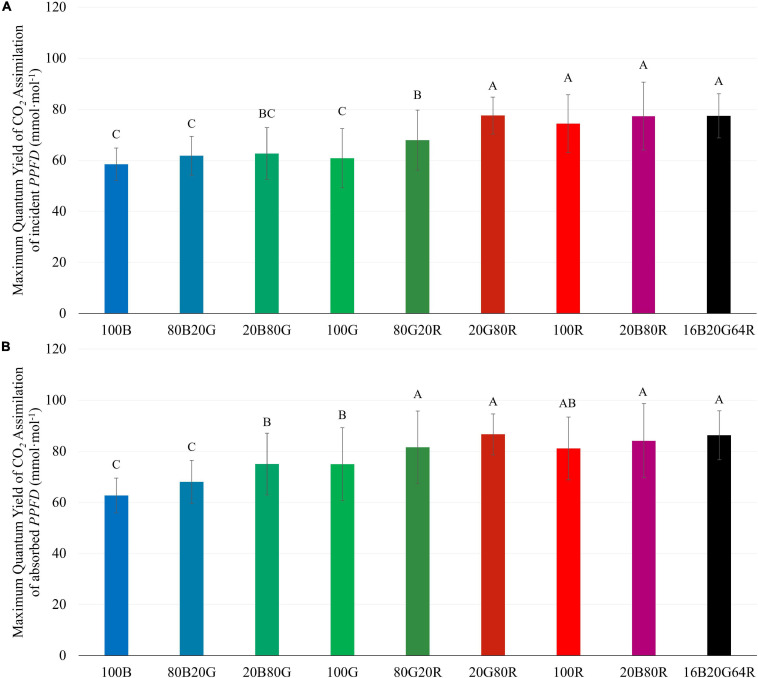
Maximum quantum yield of CO_2_ assimilation of “Green Towers” lettuce based on incident (*QY*_m,inc_) **(A)** and absorbed light (*QY*_m,abs_) **(B)** under nine different light spectra. Values are calculated as the initial slope of the light response curves of corresponding light spectra (see Figure 3). Bars with the same letter are not statistically different (*p* ≤ 0.05). Error bars represent the standard deviation (*n* = 9). The composition of the nine light spectra is shown in Table 1.

The *QY*_m,inc_ of lettuce plants was 22 and 27% higher under red light (74.3 mmol⋅mol^–1^) than under either 100G (60.8 mmol⋅mol^–1^) or 100B (58.4 mmol⋅mol^–1^), respectively (Figure 4A and Supplementary Table 1). Spectra with a high fraction of red light (64% or more) resulted in a high *QY*_m,inc_ (Figure 4A), while 80G20R resulted in an intermediate *QY*_m,inc_ (Figure 4A). To determine whether differences in *QY*_m,inc_ were due to differences in absorptance or in the ability of plants to use the absorbed photons for CO_2_ assimilation, we also calculated *QY*_m,abs_. On an absorbed light basis, 100B light still resulted in the lowest *QY*_m,abs_ (62.7 mmol⋅mol^–1^) and red light resulted in the highest *QY*_m,abs_ (81.1 mmol⋅mol^–1^) among narrow waveband lights (Figure 4B). Green light resulted in a *QY*_m,abs_ (74.9 mmol⋅mol^–1^) similar to that under red light, but significantly higher than that of blue light (Figure 4B). We did not find any interactions (synergism or antagonism) between lights of different colors, with all physiological responses under mixed spectra being similar to the weighted average of responses under single colors. Thus, for the rest of the results we focus on the three narrow waveband spectra.

**FIGURE 5:**
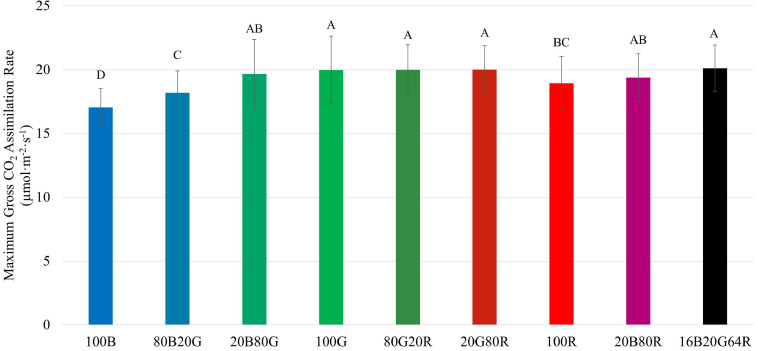
Maximum gross assimilation rate (*A*_g,max_) of “Green Towers” lettuce under different light spectra, calculated from the light response curves. Bars with the same letter are not statistically different (*p* ≤ 0.05). Error bars represent standard deviation (*n* = 9). The composition of the nine light spectra is shown in Table 1.

Among the three narrow waveband lights, 100G resulted in the highest *A*_g,max_ (20.0 μmol⋅m^–2^⋅s^–1^), followed by red (18.9 μmol⋅m^–2^⋅s^–1^), and blue light (17.0 μmol⋅m^–2^⋅s^–1^) (Figure 5 and Supplementary Table 1). As with *QY_m,inc_* and *QY*_m,abs_, combining two or three colors of light resulted in an *A*_g,max_ similar to the weighted averages of individual light colors.

**FIGURE 6:**
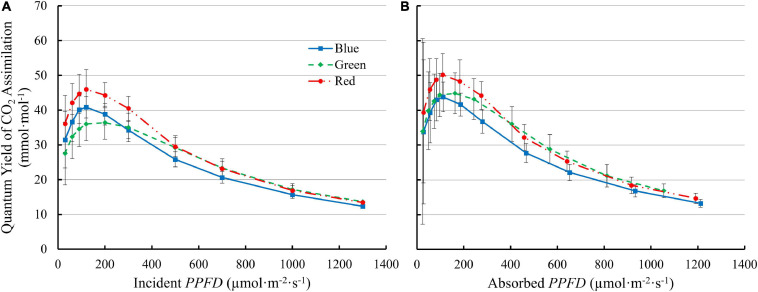
The quantum yield of CO_2_ assimilation of “Green Towers” lettuce as a function of incident (*QY*_inc_) **(A)** and absorbed *PPFD* (*QY*_abs_) **(B)** under blue, green, and red LED light. Error bars represent the standard deviation (*n* = 9).

*QY*_inc_ initially increased with increasing *PPFD* and peaked at 90–200 μmol⋅m^–2^⋅s^–1^, then decreased at higher *PPFDs* (Figure 6A). The *QY*_inc_ under 100R was higher than under either green or blue light at low *PPFD* (≤300 μmol⋅m^–2^⋅s^–1^). Although 100G resulted in lower *QY*_inc_ than 100B at low *PPFD* (≤300 μmol⋅m^–2^⋅s^–1^), the decrease in *QY*_inc_ under 100G with increasing *PPFD* was slower than that with 100B or 100R. Above 500 μmol m^–2^ s^–1^, the *QY*_inc_ with 100G was comparable to the *QY*_inc_ with 100R, and higher than with 100B (Figure 6A). The *QY*_abs_ with 100R was higher than that with either 100G or 100B at *PPFDs* from 60 to 120 μmol⋅m^–2^⋅s^–1^ (*p* < 0.05). The *QY*_abs_ with 100G was similar to 100B at low *PPFD*, but decreased slower than that with either 100R or 100B as *PPFD* increased. At *PPFD* ≥ 500 μmol⋅m^–2^⋅s^–1^, *QY*_abs_ was lowest under 100B among the three monochromatic lights (*p* < 0.05) (Figure 6B).

**FIGURE 7:**
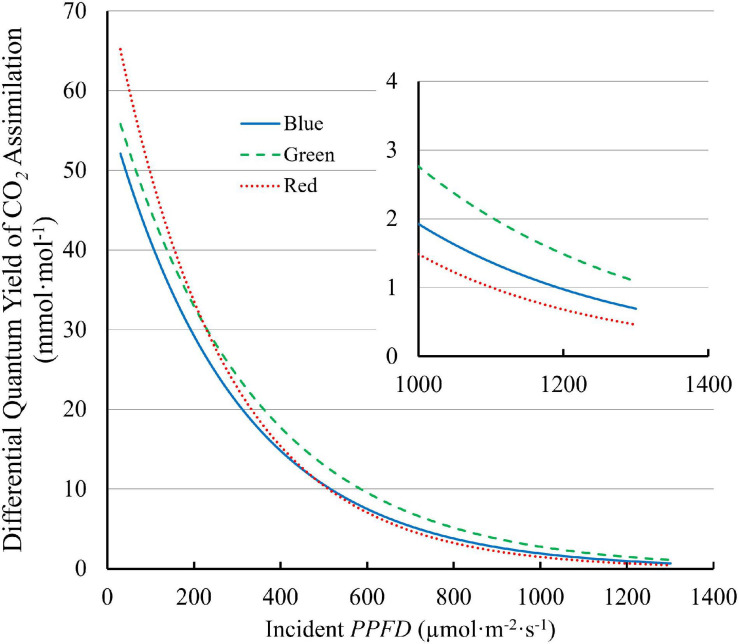
The differential quantum yield of CO_2_ assimilation (*differential QY*) of “Green Towers” lettuce under blue, green, and red LED light as a function of the *PPFD*. The *differential QY* is the increase in net assimilation per unit additional *PPFD* and was calculated as the first derivate of the light response curves (Figure 3). The insert shows the differential quantum yield plotted at *PPFDs* of 1,000–1,300 μmol m^–2^ s^–1^s to better show differences at high *PPFD* (note the different *y*-axis scale).

The differential *QY*, which quantifies the increase in CO_2_ assimilation per unit of additional *PPFD*, decreased with increasing *PPFD*. The differential *QY* with 100R was higher than those with 100B and 100G at low *PPFD*. At a *PPFD* of 30 μmol⋅m^–2^⋅s^–1^, the differential *QY* was 70.5 mmol⋅mol^–1^ for 100R, 59.4 mmol⋅mol^–1^ for 100G, and 55.8 mmol⋅mol^–1^ for 100B (Figure 7). However, the differential *QY* with 100R decreased rapidly with increasing *PPFD* and was lower than the differential *QY* with 100G at high *PPFD* (Figure 7). At high *PPFD*, the differential *QY* with 100G was highest among three monochromatic light (Figure 7). For instance, at a *PPFD* of 1,300 μmol⋅m^–2^⋅s^–1^, the differential *QY* with 100G was 1.09 mmol⋅mol^–1^, while those with 100B and 100R were 0.64 mmol⋅mol^–1^ and 0.46 mmol⋅mol^–1^, respectively (Figure 7).

### Effect of Light Spectrum and Intensity on *J* and *V*_c,max_

**FIGURE 8:**
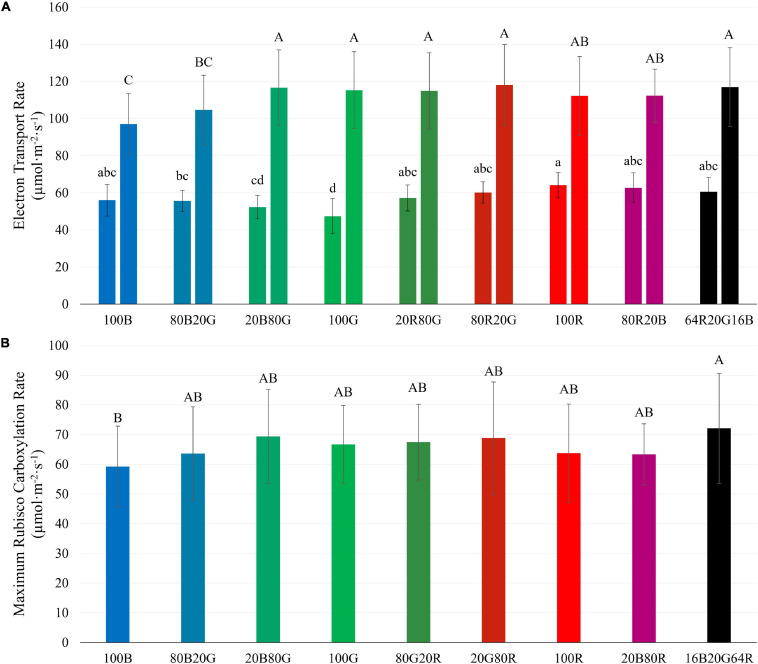
Electron transport rate (*J*) at *PPFD*s of 200 (left bars) and 1,000 μmol m^–2^ s^–1^ (right bars) **(A)** and maximum Rubisco carboxylation rate (*V*_c,max_) at a *PPFD* of 1,000 μmol m^–2^ s^–1^ **(B)** of “Green Towers” lettuce, as estimated from *A/C_i_* curves under different light spectra. Bars with the same letter are not statistically different (*p* ≤ 0.05). Error bars represent the standard deviation (*n* = 9). The light composition of the nine light spectra is shown in Table 1.

*J* of lettuce leaves at low *PPFD* was lowest under 100G (47.4 μmol⋅m^–2^⋅s^–1^), followed by 100B (56.1 μmol⋅m^–2^⋅s^–1^), and highest under 100R (64.1 μmol⋅m^–2^⋅s^–1^) (Figure 8A). At high *PPFD*, on the other hand, *J* of leaves exposed to 100G (115.3 μmol⋅m^–2^⋅s^–1^) and 100R (112.1 μmol⋅m^–2^⋅s^–1^) were among the highest, while *J* of leaves under 100B was the lowest (97.0 μmol⋅m^–2^⋅s^–1^) (Figure 8A). At high *PPFD*, *V*_c,max_ of leaves under blue light (59.3 μmol⋅m^–2^⋅s^–1^) was lower than *V*_c,max_ of leaves under 16B20G64R light (72.1 μmol⋅m^–2^⋅s^–1^), but none of the other treatments differed significantly (Figure 8). When *PPFD* increased from 200 to 1,000 μmol⋅m^–2^⋅s^–1^, *J* under green light increased by 143%, while *J* under blue and red light increased by 73% and 75%, respectively (Figure 8A). *J* and *V*_c,max_ at high *PPFD* were strongly correlated (*R*^2^ = 0.82) (Supplementary Figure 3).

## Discussion

### Interactive Effect of Light Spectrum and *PPFD* on Photosynthesis

There was an interactive effect of light spectrum and *PPFD* on photosynthetic properties of lettuce. Under low light conditions (≤200 μmol⋅m^–2^⋅s^–1^), the *QY*_inc_ of lettuce leaves under green light was lowest among blue, green, and red light (Figure 6A), due to its lower absorptance by lettuce leaves. After accounting for absorptance, green photons were used at similar efficiency as blue photons, while red photons were used most efficiently (Figure 6B). The *QY*_m,abs_ under green and red light were higher than under blue light (Figure 4B). At high *PPFD*, green and red light had similar quantum yield, higher than that of blue light, both on an absorbed and incident light basis (Figure 6A). Multiple factors contributed to the interactive effect of light spectrum and *PPFD* on quantum yield and photosynthesis.

#### Light Absorptance and Non-Photosynthetic Pigments Determine Assimilation at Low *PPFD*

*QY*_m,inc_ with blue and green light was lower than with red light (Figure 4A), consistent with McCree’s action spectrum (McCree, 1971). But when taking leaf absorptance into account, *QY*_m,abs_ was similar under green and red light and lower under blue light (Figure 4B). Similarly, at low *PPFD* (≤200 μmol⋅m^–2^⋅s^–1^), *QY*_inc_ of lettuce leaves was highest under red, intermediate under blue, and lowest under green light. When accounting for leaf absorptance, *QY*_abs_ under red light remained highest and *QY*_abs_ under both green and blue light were similar at low *PPFD* (Figure 6A). Consistent with our data, previous studies also documented that, once absorbed, green light can drive photosynthesis efficiently at low *PPFD* (Balegh and Biddulph, 1970; McCree, 1971; Evans, 1987; Sun et al., 1998; Nishio, 2000; Terashima et al., 2009; Hogewoning et al., 2012; Vogelmann and Gorton, 2014). For example, the *QY*_m,abs_ of spinach (*Spinacia oleracea*) and cabbage (*Brassica oleracea L.*) was highest under red light, followed by that under green light and lowest with blue light. But on incident light basis, *QY*_m,inc_ of under green light was lower than under red or blue light (Sun et al., 1998).

Both our data (Figure 4B) and those of Sun et al. (1998) show that *QY*_m,abs_ with blue light is lower than that with red and green light, indicating that blue light is used intrinsically less efficiently by lettuce. Blue light, and, to a lesser extent, green light is absorbed not just by chlorophyll, but also by flavonoids and carotenoids (Sun et al., 1998). Those pigments can divert energy away from photochemistry and thus reduce the *QY*_abs_ under blue light. Flavonoids (e.g., anthocyanins) are primarily located in the vacuole and cannot transfer absorbed light energy to photosynthetic pigments (Sun et al., 1998). Likewise, free carotenoids do not contribute to photochemistry (Hogewoning et al., 2012). Carotenoids in light-harvesting antennae and reaction centers channel light energy to photochemistry, but with lower transfer efficiency than chlorophylls (Croce et al., 2001; de Weerd et al., 2003a, b; Wientjes et al., 2011; Hogewoning et al., 2012). Therefore, absorption of blue light by flavonoids and carotenoids reduces the quantum yield of CO_2_ assimilation. Thus, even with the high absorptance of blue light by green leaves, *QY*_m,abs_ of leaves under blue light was the lowest among the three monochromatic lights (Figure 4B). It is likely that the lower *QY*_abs_ under green light than that under red light was also due to absorption of green light by carotenoids and flavonoids (Hogewoning et al., 2012). At high *PPFD*, absorption of blue light by flavonoids and carotenoids still occurs, but this is less of a limiting factor for photosynthesis, since light availability is not limiting under high *PPFD*.

#### Light Dependence of Respiration and Rubisco Activity May Reduce the Quantum Yield at Low *PPFD*

At *PPFD*s below 200 μmol⋅m^–2^⋅s^–1^, the *QY*_inc_ and *QY*_abs_ of lettuce showed an unexpected pattern in response to *PPFD* (Figure 6). Unlike the quantum yield of PSII, which decreases exponentially with increasing *PPFD* (Weaver and van Iersel, 2019), *QY*_inc_ and *QY*_abs_ increased initially with increasing *PPFD* (Figure 6). A similar pattern was previously observed by Craver et al. (2020) in petunia (*Petunia* × *hybrida*) seedlings. This pattern could result from light-dependent regulation of respiration (Croce et al., 2001), alternative electron sinks such as nitrate reduction (Skillman, 2008; Nunes-Nesi et al., 2010), or Rubisco activity (Campbell and Ogren, 1992; Zhang and Portis, 1999). In our calculations, we assumed that the leaf respiration in the light was the same as *R*_d_. However, leaf respiration in the light is lower than in the dark, in a *PPFD*-dependent manner (Brooks and Farquhar, 1985; Atkin et al., 1997), which can lead to overestimation of *A*_g_ with increasing *PPFD*. When we accounted for this down-regulation of respiration, using the model by Müller et al. (2005) to correct *A*_g_, *QY_inc_*, and *QY*_abs_, we found that depression of respiration by light did not explain the initial increase in *QY*_inc_ and *QY*_abs_ we observed (Supplementary Figure 4). Alternative electron sinks in the chloroplasts that are upregulated in response to light can explain the low *QY_inc_*, and *QY*_abs_ at low *PPFD*, because they compete with the Calvin cycle for reducing power (ferredoxin/NADPH). Such processes include photorespiration (Krall and Edwards, 1992), nitrate assimilation (Nunes-Nesi et al., 2010), sulfate assimilation (Takahashi et al., 2011) and the Mehler reaction (Badger et al., 2000) and their effect on *QY_inc_*, and *QY*_abs_ would be especially notable under low *PPFD* (Supplementary Figure 5).

Upregulation of Rubisco activity by Rubisco activase in the light may also have contributed to the increase in *QY*_inc_ and *QY*_abs_ at low *PPFD* (Campbell and Ogren, 1992; Zhang and Portis, 1999). In the dark, 2-carboxy-D-arabinitol-1-phosphate (CA1P) or RuBP binds strongly to the active sites of Rubisco, preventing carboxylation activity. In the light, Rubisco activase releases the inhibitory CA1P or RuBP from the catalytic site of Rubisco, in a light-dependent manner (Campbell and Ogren, 1992; Zhang and Portis, 1999; Parry et al., 2008). At *PPFD* < 120 μmol⋅m^–2^⋅s^–1^, low Rubisco activity may have limited photosynthesis.

#### Light Distribution Within Leaves Affects *QY* at High *PPFD*

Except for the initial increase at low *PPFD*, both *QY*_inc_ and *QY*_abs_ decreased with increasing *PPFD*. *QY*_inc_ decreased slower under green than under red or blue light (Figure 6A). At a *PPFD* ≥ 500 μmol⋅m^–2^⋅s^–1^, *QY*_inc_ under green light was higher than that under blue light (Figure 6A). Accordingly, *A*_n_ under blue light was lower than under green and red light at *PPFD*s above 500 μmol⋅m^–2^⋅s^–1^ (Figure 3A). The lower *QY*_inc_ under blue light than under green and red light at high *PPFD* can be explained by disparities in the light distribution within leaves.

Blue and red light were strongly absorbed by lettuce leaves (93.2 and 91.6%, respectively), while green light was absorbed less (81.1%) (Table 2). Similar low green absorptance was found in sunflower (*Helianthus annuus* L.), snapdragon (*Antirrhínum majus* L.) (Brodersen and Vogelmann, 2010), and spinach (Vogelmann and Han, 2000). In leaves of those species, absorption of red and blue light peaked in the upper 20% of leaves, and declined sharply further into the leaf. Absorption of red light decreased slower with increasing depth than that of blue light (Vogelmann and Han, 2000; Brodersen and Vogelmann, 2010). Green light absorption peaked deeper into leaves, and was more evenly distributed throughout leaves, because of low absorption of green light by chlorophyll (Vogelmann and Han, 2000; Brodersen and Vogelmann, 2010). The more even distribution of green light within leaves, as compared to red and blue light, can explain the interactive effects between *PPFD* and light spectrum on leaf photosynthesis. It was estimated that less than 10% of blue light traveled through the palisade mesophyll and reached the spongy mesophyll in spinach, while about 35% of green light and 25% of red light did so (Vogelmann and Evans, 2002). It was also estimated that chlorophyll in the lowermost chloroplasts of spinach leaves absorbed about 10% of green and <2% of blue light, compared to chlorophyll in the uppermost chloroplasts (Vogelmann and Evans, 2002; Terashima et al., 2009).

The more uniform green light distribution within leaves may be a key contributor to higher leaf level *QY*_inc_ under high *PPFD* because less heat dissipation of excess light energy is needed (Nishio, 2000; Terashima et al., 2009). Reaction centers near the adaxial leaf surface receive more excitation energy under blue, and to a lesser extent under red light, than under green light, because of the differences in absorptance. Consequently, under high intensity blue light, NPQ is up-regulated in the chloroplasts near the adaxial leaf surface to dissipate some of the excitation energy (Sun et al., 1998; Nishio, 2000), lowering the *QY*_inc_ under blue light. Since less green light is absorbed near the adaxial surface, less heat dissipation is required. When incident light increased from 150 to 600 μmol⋅m^–2^⋅s^–1^, the fraction of whole leaf CO_2_ assimilation that occurred in the top half of spinach leaves remained the same under green light (58%), but decreased from 87 to 73% under blue light. This indicates more upregulation of heat dissipation in the top of the leaves under blue, than under green light (Evans and Vogelmann, 2003). On the other hand, the bottom half of the leaves can still utilize the available light with relatively high *QY*_inc_, since the amount of light reaching the bottom half is relatively low, even under high *PPFD* (Nishio, 2000). By channeling more light to the under-utilized bottom part of leaves, leaves could achieve higher *QY*_inc_ even under high intensity green light. In our study, high *QY*_inc_ under green light and low *QY*_inc_ under blue light at high *PPFD* (Figure 6) can be thus explained by the large disparities in the light environment in chloroplasts from the adaxial to the abaxial side of leaves due to differences in leaf absorptance. Similarly, differential *QY* of lettuce leaves was highest under green light and lower under blue and red light at high *PPFD* (>300 μmol⋅m^–2^⋅s^–1^) (Figure 7), also potentially because of the more uniform distribution of green light and the uneven distribution of blue and red light in leaves.

Along the same line, *A*_n_ of lettuce leaves was the lowest under blue light at *PPFD* > 500 μmol⋅m^–2^⋅s^–1^ (Figure 3). Also, *A*_n_ of lettuce leaves approached light saturation at lower *PPFD*s under blue and red light, than under green light (Figure 3A). Under blue, green, and red light, lettuce leaves reached 95% of *A*_n,max_ at *PPFD*s of 954, 1,110 and 856 μmol⋅m^–2^⋅s^–1^, respectively. This can be seen more clearly in the differential *QY* at high *PPFD* (Figure 7). At a *PPFD* of 1,300 μmol⋅m^–2^⋅s^–1^, green light had a differential *QY* of 1.09 mmol⋅mol^–1^, while that of red and blue light was only 0.46 and 0.69 mmol⋅mol^–1^, respectively (Figure 7). Green light also resulted in a higher *A*_g,max_ (22.9 μmol⋅m^–2^⋅s^–1^) than red and blue light (21.8 and 19.3 μmol⋅m^–2^⋅s^–1^, respectively) (Figure 5). As discussed before, the high *A*_g,max_ under green light resulted from the more uniform light distribution under green light, allowing deeper cell layers to photosynthesize more. Previous research similarly found that at high *PPFD* (>500 μmol⋅m^–2^⋅s^–1^), *A*_n_ of both spinach and cabbage were lower under blue light than under white, red and green light (Sun et al., 1998). Overall, under high *PPFD*, the differences in light distribution throughout a leaf are important to quantum yield and assimilation rate, since it affects NPQ up-regulation (Sun et al., 1998; Nishio, 2000). However, light distribution within a leaf is less important at low than at high *PPFD*, because upregulation of NPQ increases with increasing *PPFD* (Zhen and van Iersel, 2017).

### Light Spectrum Affects *J*, but Not *V*_c,max_

We examined the effect of light quality and intensity on *J* and *V*_c,max_ (Figure 8). For the light-dependent reactions, the interactive effect between light spectra and *PPFD* found for CO_2_ assimilation and quantum yield was also observed for *J* (Figure 8A). At low *PPFD* (200 μmol⋅m^–2^⋅s^–1^), green light resulted in the lowest *J* and red light in the highest *J* among single waveband spectra. But at a *PPFD* of 1,000 μmol⋅m^–2^⋅s^–1^, red and green light resulted in the highest *J* and blue light in the lowest *J* (Figure 8A), similar to the differences in *A*_g_.

There was no clear evidence of Rubisco limitations to photosynthesis at a *PPFD* of 200 μmol⋅m^–2^⋅s^–1^, so the rate of the light-dependent reactions likely limited photosynthesis. This is corroborated by the strong correlation between *A*_g_ and *J* at a *PPFD* of 200 μmol⋅m^–2^⋅s^–1^. Although Rubisco limitations to photosynthesis were observed at a *PPFD* of 1,000 μmol⋅m^–2^⋅s^–1^, there were no meaningful differences in *V*_c,max_ in response to light spectrum, in contrast to *J* (Figure 8).

When *PPFD* increased 5×, from 200 to 1,000 μmol⋅m^–2^⋅s^–1^, there was only a 1.7 to 2.4× increase in *J*, indicating a lower *QY(J)_inc_* at higher *PPFD*. This matches the lower *QY*_inc_ and the asymptotic increase in *A*_n_ in response to increasing *PPFD* (Figure 3). The relative increase of *J* under green light (143%) was greater than that under both blue and red light (73 and 75%, respectively) as *PPFD* increased. This similarly can be attributed to a more uniform energy distribution of green light among reaction centers throughout a leaf and weaker upregulation of non-photochemical quenching with increasing green light intensity (Sun et al., 1998; Nishio, 2000; Evans and Vogelmann, 2003), as discussed before.

**FIGURE 9:**
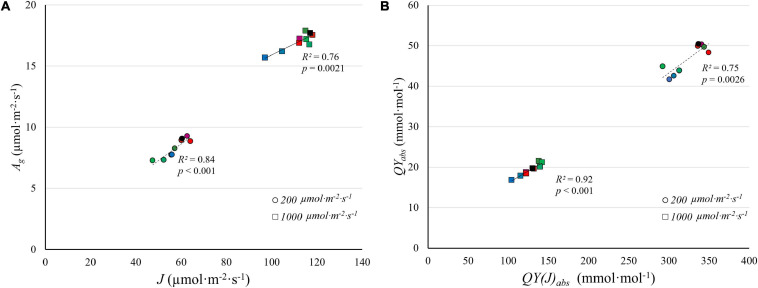
The correlation between gross CO_2_ assimilation rate (*A*_g_) estimated from light response curves and electron transport rate (*J*) estimated from *A/C_i_* curves **(A)**, and between the quantum yield of CO_2_ assimilation (*QY*_abs_) and the quantum yield of electron transport on an absorbed light basis [*QY(J)_abs_*] **(B)**, under low *PPFD* (200 μmol m^–2^ s^–1^) and high *PPFD* (1,000 μmol m^–2^ s^–1^) under nine light spectra averaged over nine “Green Towers” lettuce plants. The color scheme representing the nine spectra is the same as Figure 8.

There was a strong correlation between *J* and *A*_g_ under the nine light spectra at both *PPFD* levels (Figure 9A). *QY*_abs_ and *QY(J)_abs_* are similarly strongly correlated (Figure 9B). Unlike *J*, *V*_c,max_ was largely unaffected by light spectra (Figure 8B) and was not correlated with *A*_g_ (data not shown). There was, however, a strong correlation between *J* and *V*_c,max_ at a *PPFD* of 1,000 μmol⋅m^–2^⋅s^–1^ (*R*^2^ = 0.82, Supplementary Figure 3), suggesting that *J* and *V*_c,max_ are co-regulated. Similarly, Wullschleger (1993) noted a strong linear relationship between *J* and *V*_c,max_ across 109 C_3_ species. The ratio between *J* and *V*_c,max_ in our study (1.5–2.0) similar to the ratio found by Wullschleger (1993). These results suggest that the interactive effect of light spectra and *PPFD* resulted from effects on *J*, which is associated with light energy harvesting by reaction centers, rather than from *V*_c,max_.

### No Interactive Effects Among Blue, Green, and Red Light

The Emerson enhancement effect describes a synergistic effect between lights of different wavebands (red and far-red) on photosynthesis (Emerson, 1957). McCree (1971) attempted to account for interactions between light with different spectra when developing photosynthetic action spectra and applied low intensity monochromatic lights from 350 to 725 nm with white background light to plants. His results showed no interactive effect between those monochromatic lights and white light (McCree, 1971). We tested different ratios of blue, green, and red light and different *PPFD*s, and similarly did not find any synergistic or antagonistic effect of different wavebands on any physiological parameters measured or calculated.

### Importance of Interactions Between *PPFD* and Light Quality and Its Applications

The interactive effect between *PPFD* and light quality demonstrates a remarkable adaptation of plants to different light intensities. By not absorbing green light strongly, plants open up a “green window,” as Terashima et al. (2009) called it, to excite chloroplasts deeper into leaves, and thus facilitating CO_2_ assimilation throughout the leaf. While red light resulted in relatively high *QY*_inc_, *QY*_abs_ and *A*_n_ at both high and low *PPFD* (Figures 3, 6), it is still mainly absorbed in the upper part of leaves (Sun et al., 1998; Brodersen and Vogelmann, 2010). Green light can penetrate deeper into leaves (Brodersen and Vogelmann, 2010) and help plants drive efficient CO_2_ assimilation at high *PPFD* (Figures 3, 5).

Many early photosynthesis studies investigated the absorptance and action spectrum of photosynthesis of green algae, e.g., Haxo and Blinks (1950) or chlorophyll or chloroplasts extracts, e.g., Chen (1952). Extrapolating light absorptance of green algae and suspension of chlorophyll or chloroplast to whole leaves from can lead to an underestimation of absorptance of green light by whole leaves and the belief that green light has little photosynthetic activity (Moss and Loomis, 1952; Smith et al., 2017). Photosynthetic action spectra developed on whole leaves of higher plants, however, have long shown that green light effectively contributes to CO_2_ assimilation, although with lower *QY*_inc_ than red light (Hoover, 1937; McCree, 1971; Inada, 1976; Evans, 1987). The importance of green light for photosynthesis was clearly established in more recent studies, emphasizing its role in more uniformly exciting all chloroplasts, which especially important under high *PPFD* (Sun et al., 1998; Nishio, 2000; Terashima et al., 2009; Hogewoning et al., 2012; Smith et al., 2017). The idea that red and blue light are more efficient at driving photosynthesis, unfortunately, still lingers, e.g., Singh et al. (2015).

Light-emitting diodes (LEDs) have received wide attention in recent years for use in controlled environment agriculture, as they now have superior efficacy over traditional lighting technologies (Pattison et al., 2018). LEDs can have a narrow spectrum and great controllability. This provides unprecedented opportunities to fine tune light spectra and *PPFD* to manipulate crop growth and development. Blue and red LEDs have higher efficacy than white and green LEDs (Kusuma et al., 2020). By coincidence, McCree’s action spectrum (Figure 1; McCree, 1971) also has peaks in the red and blue region, although the peak in the blue region is substantially lower than the one in the red region. Therefore, red and blue LEDs are sometimes considered optimal for driving photosynthesis. This claim holds true only under low *PPFD*. Green light plays an important role in photosynthesis, as it helps plants to adapt to different light intensities. The wavelength-dependent absorptance of chlorophylls channels green light deeper into leaves, resulting in more uniform light absorption throughout leaves and providing excitation energy to cells further from the adaxial surface. Under high *PPFD*, this can increase leaf photosynthesis. Plant evolved under sunlight for hundreds of millions of years, and it seems likely that the relatively low absorptance of green light contributes to the overall photosynthetic efficiency of plants (Nishio, 2000).

## Conclusion

There was an interactive effect of light spectrum and *PPFD* on leaf photosynthesis. Under low *PPFD*, *QY*_inc_ was lowest under green and highest under red light. The low *QY*_inc_ under green light at low *PPFD* was due to low absorptance. In contrast, at high *PPFD*, green and red light achieved similar *QY*_inc_, higher than that of blue light. The strong absorption of blue light by chlorophyll creates a large light gradient from the top to the bottom of leaves. The large amount of excitation energy near the adaxial side of a leaf results in upregulation of nonphotochemical quenching, while chloroplasts near the bottom of a leaf receive little excitation energy under blue light. The more uniform distribution of green light absorption within leaves reduces the need for nonphotochemical quenching near the top of the leaf, while providing more excitation energy to cells near the bottom of the leaf. We also found that the interactive effect of light spectrum and *PPFD* on photosynthesis was a result of the light-dependent reactions; gross assimilation and *J* were strongly correlated. We detected no synergistic or antagonistic interactions between blue, green, and red light.

## Data Availability Statement

The raw data supporting the conclusions of this article will be made available by the authors, without undue reservation.

## Author Contributions

JL and MI designed the experiment, discussed the data, and revised the manuscript. JL performed the experiment, analyzed data, and prepared the first draft. Both authors contributed to the article and approved the submitted version.

## Conflict of Interest

The authors declare that the research was conducted in the absence of any commercial or financial relationships that could be construed as a potential conflict of interest.

## Supplementary Material

Supplementary Figure 1 (Related to Figure 6) Quantum yield of CO_2_ assimilation of “Green Towers” lettuce as a function of incident (*QY*_inc_) **(A,C,E,G)** and absorbed *PPFD* (*QY*_abs_) **(B,D,F,H)** under nine light spectra (see Table 1). Error bars represent standard deviation (*n* = 9).

Supplementary Figure 2 (Related to Figure 7) Differential quantum yield of CO_2_ assimilation (*differential QY*) of “Green Towers” lettuce under nine light spectra as a function of the *PPFD*. Inserts show *differential QY* at *PPFD*s of 1,000–1,300 μmol⋅m^–2^ s^–1^s to better show differences at high *PPFD* (note the different *y*-axis scale). The composition of the nine light spectra is shown in Table 1. The light spectra in the graphs are **(A)** 100B, 100G and 100R; **(B)** 100B, 80B20G, 20B80G and 100G; **(C)** 100G, 80G20R, 20G80R and 100R; and **(D)** 20B80R, 16B20G64R and 100G.

Supplementary Figure 3 (Related to Figure 6) The correlation between electron transport (*J*) and maximum Rubisco carboxylation rate (*V*_c,max_) of “Green Towers” lettuce estimated from *A/C_i_* curves under *PPFD* (1000 μmol m^–2^ s^–1^) under nine light spectra (*p* < 0.001).

Supplementary Figure 4 (Related to Figure 6) The comparison between *QY*_inc_ before **(A)** and after **(B)** correcting for light-suppression of respiration under blue, green, and red LED light. Note that the initial increase in *QY*_inc_ became more pronounced after correction of light suppressed respiration.

Supplementary Figure 5 The comparison between *QY*_abs_ before **(A)** and after **(B)** correcting for alternative electron sinks under blue, green, and red LED light. Assuming a simplified electron sink that diverts energy of 15 μmol m^–2^ s^–1^ of absorbed photons (an arbitrary value used for illustrative purposes only) away from the Calvin cycle under all *PPFD*s, the corrected *QY*_abs_ was calculated based on remaining photons available to support Calvin cycle processes **(B)**. Note that the pattern of *QY*_inc_ after correcting of alternative electron sink **(B)** is similar to quantum yield of PSII measured by chlorophyll fluorescence by Weaver and van Iersel (2019).

Supplementary Table 1 Dark respiration rate (R_d_), maximum quantum yield of CO_2_ assimilation (QY_m,inc_) and maximum gross assimilation rate (A_g,max_) of “Green towers” lettuce derived from the light response curves for nine different spectra using Eq. 1. The light response curves are shown in Figure 3. *See light composition of nine lights presented here in Table 1.

## Abbreviations

*PPFD*: photosynthetic photon flux density
RuBP: ribulose 1,5-bisphosphate
Rubisco: ribulose-1,5-bisphosphate carboxylase/oxygenase
VPD: vapor pressure deficit
FWHM: full width at half maximum
*A*_n_: net CO_2_ assimilation rate
*R*_d_: dark respiration rate
*QY*_m,inc_: maximum quantum yield of CO_2_ assimilation
*A*_g,max_: light-saturated gross assimilation rate
*QY*_m,abs_: maximum quantum yield of CO_2_ assimilation on absorbed light base
*QY*_inc_: quantum yield of CO_2_ assimilation based on incident *PPFD*
*A*_g_: gross CO_2_ assimilation rate
*QY*_abs_: quantum yield of CO_2_ assimilation on absorbed light base
*QY*: quantum yield of CO_2_ assimilation
*A/Ci* curve: assimilation – internal leaf CO_2_ response curve
RACiR: rapid *A/C_i_* response technique
*V*_c,max_: maximum rate of Rubisco carboxylation
*J*: rate of electron transport
CA1P: 2-carboxy-D-arabinitol-1-phosphate
NPQ: non-photochemical quenching.

## References

[B1] Atkin O. K. Westbeek M. Cambridge M. L. Lambers H. Pons T. L. (1997). Leaf respiration in light and darkness (A comparison of slow- and fast-growing *Poa* species). *Plant Physiol.* 113 961–965. 10.1104/pp.113.3.961 12223656 PMC158216

[B2] Badger M. R. von Caemmerer S. Ruuska S. Nakano H. (2000). Electron flow to oxygen in higher plants and algae: rates and control of direct photoreduction (Mehler reaction) and rubisco oxygenase. *Philos. Trans. R. Soc. Lond. B Biol. Sci.* 355 1433–1446. 10.1098/rstb.2000.0704 11127997 PMC1692866

[B3] Balegh S. E. Biddulph O. (1970). The photosynthetic action spectrum of the bean plant. *Plant Physiol.* 46 1–5. 10.1104/pp.46.1.1 16657397 PMC396523

[B4] Brodersen C. R. Vogelmann T. C. (2010). Do changes in light direction affect absorption profiles in leaves? *Funct. Plant Biol.* 37 403–412. 10.1071/fp09262

[B5] Brooks A. Farquhar G. D. (1985). Effect of temperature on the CO2/O2 specificity of ribulose-1,5-bisphosphate carboxylase/oxygenase and the rate of respiration in the light. *Planta* 165 397–406. 10.1007/BF00392238 24241146

[B6] Campbell W. J. Ogren W. L. (1992). Light activation of rubisco by rubisco activase and thylakoid membranes. *Plant Cell Physiol.* 33 751–756. 10.1093/oxfordjournals.pcp.a078314

[B7] Chen S. L. (1952). The action spectrum for the photochemical evolution of oxygen by isolated chloroplasts. *Plant Physiol.* 27 35–48. 10.1104/pp.27.1.35 16654441 PMC540303

[B8] Craver J. K. Nemali K. S. Lopez R. G. (2020). Acclimation of growth and photosynthesis in *Petunia* seedlings exposed to high-intensity blue radiation. *J. Am. Soc. Hortic. Sci.* 145 152–161. 10.21273/jashs04799-19

[B9] Croce R. Müller M. G. Bassi R. Holzwarth A. R. (2001). Carotenoid-to-chlorophyll energy transfer in recombinant major light-harvesting complex (LHCII) of higher plants. I. Femtosecond transient absorption measurements. *Biophys. J.* 80 901–915. 10.1016/S0006-3495(01)76069-9 11159457 PMC1301288

[B10] de Weerd F. L. Dekker J. P. van Grondelle R. (2003a). Dynamics of β-carotene-to-chlorophyll singlet energy transfer in the core of photosystem II. *J. Phys. Chem. B* 107 6214–6220. 10.1021/jp027737q

[B11] de Weerd F. L. Kennis J. T. Dekker J. P. van Grondelle R. (2003b). β-Carotene to chlorophyll singlet energy transfer in the photosystem I core of *Synechococcus elongatus* proceeds via the β-carotene S2 and S1 states. *J. Phys. Chem. B* 107 5995–6002. 10.1021/jp027758k

[B12] Emerson R. (1957). Dependence of yield of photosynthesis in long-wave red on wavelength and intensity of supplementary light. *Science* 125 746–746. 10.1126/science.125.3251.746 17731423

[B13] Evans J. (1987). The dependence of quantum yield on wavelength and growth irradiance. *Funct. Plant Biol.* 14 69–79. 10.1071/PP9870069

[B14] Evans J. Vogelmann T. C. (2003). Profiles of 14C fixation through spinach leaves in relation to light absorption and photosynthetic capacity. *Plant Cell Environ.* 26 547–560. 10.1046/j.1365-3040.2003.00985.x

[B15] Haxo F. T. Blinks L. (1950). Photosynthetic action spectra of marine algae. *J. Gen. Physiol.* 33 389–422. 10.1085/jgp.33.4.389 15406376 PMC2147193

[B16] Hogewoning S. W. Wientjes E. Douwstra P. Trouwborst G. Van Ieperen W. Croce R. (2012). Photosynthetic quantum yield dynamics: from photosystems to leaves. *Plant Cell* 24 1921–1935. 10.1105/tpc.112.097972 22623496 PMC3442578

[B17] Hoover W. H. (1937). The dependence of carbon dioxide assimilation in a higher plant on wave length of radiation. *Smithson. Misc. Collect.* 95 1–13.

[B18] Inada K. (1976). Action spectra for photosynthesis in higher plants. *Plant Cell Physiol.* 17 355–365. 10.1093/oxfordjournals.pcp.a075288

[B19] Krall J. P. Edwards G. E. (1992). Relationship between photosystem II activity and CO2 fixation in leaves. *Physiol. Plant.* 86 180–187. 10.1111/j.1399-3054.1992.tb01328.x

[B20] Kusuma P. Pattison P. M. Bugbee B. (2020). From physics to fixtures to food: current and potential LED efficacy. *Hortic. Res.* 7:56. 10.1038/s41438-020-0283-7 32257242 PMC7105460

[B21] McCree K. J. (1971). The action spectrum, absorptance and quantum yield of photosynthesis in crop plants. *Agric. Meteorol.* 9 191–216. 10.1016/0002-1571(71)90022-7

[B22] McCree K. J. (1972). Test of current definitions of photosynthetically active radiation against leaf photosynthesis data. *Agric. Meteorol.* 10 443–453. 10.1016/0002-1571(72)90045-3

[B23] Moss R. A. Loomis W. E. (1952). Absorption spectra of leaves. I. the visible spectrum. *Plant Physiol.* 27 370–391. 10.1104/pp.27.2.370 16654461 PMC540339

[B24] Müller J. Wernecke P. Diepenbrock W. (2005). LEAFC3-N: a nitrogen-sensitive extension of the CO_2_ and H_2_O gas exchange model LEAFC3 parameterised and tested for winter wheat (Triticum aestivum L.). *Ecol. Modell.* 183 183–210. 10.1016/j.ecolmodel.2004.07.025

[B25] Nishio J. (2000). Why are higher plants green? Evolution of the higher plant photosynthetic pigment complement. *Plant Cell Environ.* 23 539–548. 10.1046/j.1365-3040.2000.00563.x

[B26] Nunes-Nesi A. Fernie A. R. Stitt M. (2010). Metabolic and signaling aspects underpinning the regulation of plant carbon nitrogen interactions. *Mol. plant* 3 973–996. 20926550 10.1093/mp/ssq049

[B27] Parry M. A. Keys A. J. Madgwick P. J. Carmo-Silva A. E. Andralojc P. J. (2008). Rubisco regulation: a role for inhibitors. *J. Exp. Bot.* 59 1569–1580. 10.1093/jxb/ern084 18436543

[B28] Pattison P. Lee K. Stober K. Yamada M. (2018). *Energy Savings Potential of SSL in Horticultural Applications.* Washington, DC: U.S. Department of Energy, Office of Scientific and Technical Information.

[B29] Sharkey T. D. Bernacchi C. J. Farquhar G. D. Singsaas E. L. (2007). Fitting photosynthetic carbon dioxide response curves for C3 leaves. *Plant Cell Environ.* 30 1035–1040. 10.1111/j.1365-3040.2007.01710.x 17661745

[B30] Singh D. Basu C. Meinhardt-Wollweber M. Roth B. (2015). LEDs for energy efficient greenhouse lighting. *Renew. Sustain. Energy Rev.* 49 139–147.

[B31] Skillman J. B. (2008). Quantum yield variation across the three pathways of photosynthesis: not yet out of the dark. *J. Exp. Bot.* 59 1647–1661. 10.1093/jxb/ern029 18359752

[B32] Smith H. L. McAusland L. Murchie E. H. (2017). Don’t ignore the green light: exploring diverse roles in plant processes. *J. Exp. Bot.* 68 2099–2110. 10.1093/jxb/erx098 28575474

[B33] Stinziano J. R. Morgan P. B. Lynch D. J. Saathoff A. J. McDermitt D. K. Hanson D. T. (2017). The rapid A–Ci response: photosynthesis in the phenomic era. *Plant Cell Environ.* 40 1256–1262. 10.1111/pce.12911 28247953

[B34] Sun J. Nishio J. N. Vogelmann T. C. (1998). Green light drives CO2 fixation deep within leaves. *Plant Cell Physiol.* 39 1020–1026. 10.1093/oxfordjournals.pcp.a029298

[B35] Takahashi H. Kopriva S. Giordano M. Saito K. Hell R. (2011). Sulfur assimilation in photosynthetic organisms: molecular functions and regulations of transporters and assimilatory enzymes. *Annu. Rev. Plant Biol.* 62 157–184. 10.1146/annurev-arplant-042110-103921 21370978

[B36] Terashima I. Fujita T. Inoue T. Chow W. S. Oguchi R. (2009). Green light drives leaf photosynthesis more efficiently than red light in strong white light: revisiting the enigmatic question of why leaves are green. *Plant cell physiol.* 50 684–697. 10.1093/pcp/pcp034 19246458

[B37] Vogelmann T. Han T. (2000). Measurement of gradients of absorbed light in spinach leaves from chlorophyll fluorescence profiles. *Plant Cell Environ.* 23 1303–1311. 10.1046/j.1365-3040.2000.00649.x

[B38] Vogelmann T. C. Evans J. (2002). Profiles of light absorption and chlorophyll within spinach leaves from chlorophyll fluorescence. *Plant Cell Environ.* 25 1313–1323. 10.1046/j.1365-3040.2002.00910.x

[B39] Vogelmann T. C. Gorton H. L. (2014). “Leaf: light capture in the photosynthetic organ,” in *The Structural Basis of Biological Energy Generation*, ed. Hohmann-Marriott M. F. (Dordrecht: Springer Netherlands), 363–377.

[B40] Weaver G. van Iersel M. W. (2019). Photochemical characterization of greenhouse-grown Lettuce (*Lactuca sativa* L. ‘Green Towers’) with applications for supplemental lighting control. *HortScience* 54 317–322. 10.21273/hortsci13553-18

[B41] Wientjes E. van Stokkum I. H. van Amerongen H. Croce R. (2011). The role of the individual Lhcas in photosystem I excitation energy trapping. *Biophys. J.* 101 745–754. 10.1016/j.bpj.2011.06.045 21806943 PMC3145314

[B42] Wullschleger S. D. (1993). Biochemical limitations to carbon assimilation in C3 Plants—a retrospective analysis of the A/Ci curves from 109 species. *J. Exp. Bot.* 44 907–920. 10.1093/jxb/44.5.907 12432039

[B43] Zhang N. Portis A. R. (1999). Mechanism of light regulation of rubisco: a specific role for the larger rubisco activase isoform involving reductive activation by thioredoxin-f. *Proc. Natl. Acad. Sci. U.S.A.* 96 9438–9443. 10.1073/pnas.96.16.9438 10430961 PMC17801

[B44] Zhen S. Haidekker M. van Iersel M. W. (2019). Far−red light enhances photochemical efficiency in a wavelength−dependent manner. *Physiol. Plant.* 167 21–33. 10.1111/ppl.12834 30203475

[B45] Zhen S. van Iersel M. W. (2017). Photochemical acclimation of three contrasting species to different light levels: implications for optimizing supplemental lighting. *J. Am. Soc. Hortic. Sci.* 142 346–354. 10.21273/jashs04188-17

