# Supplementary figures and images for: Photosynthetic Physiology of Blue, Green, and Red Light: Light Intensity Effects and Underlying Mechanisms
Source: Front Plant Sci. 2021 Mar 5;12:619987. doi: 10.3389/fpls.2021.619987 (PMC7977723; doi:10.3389/fpls.2021.619987)

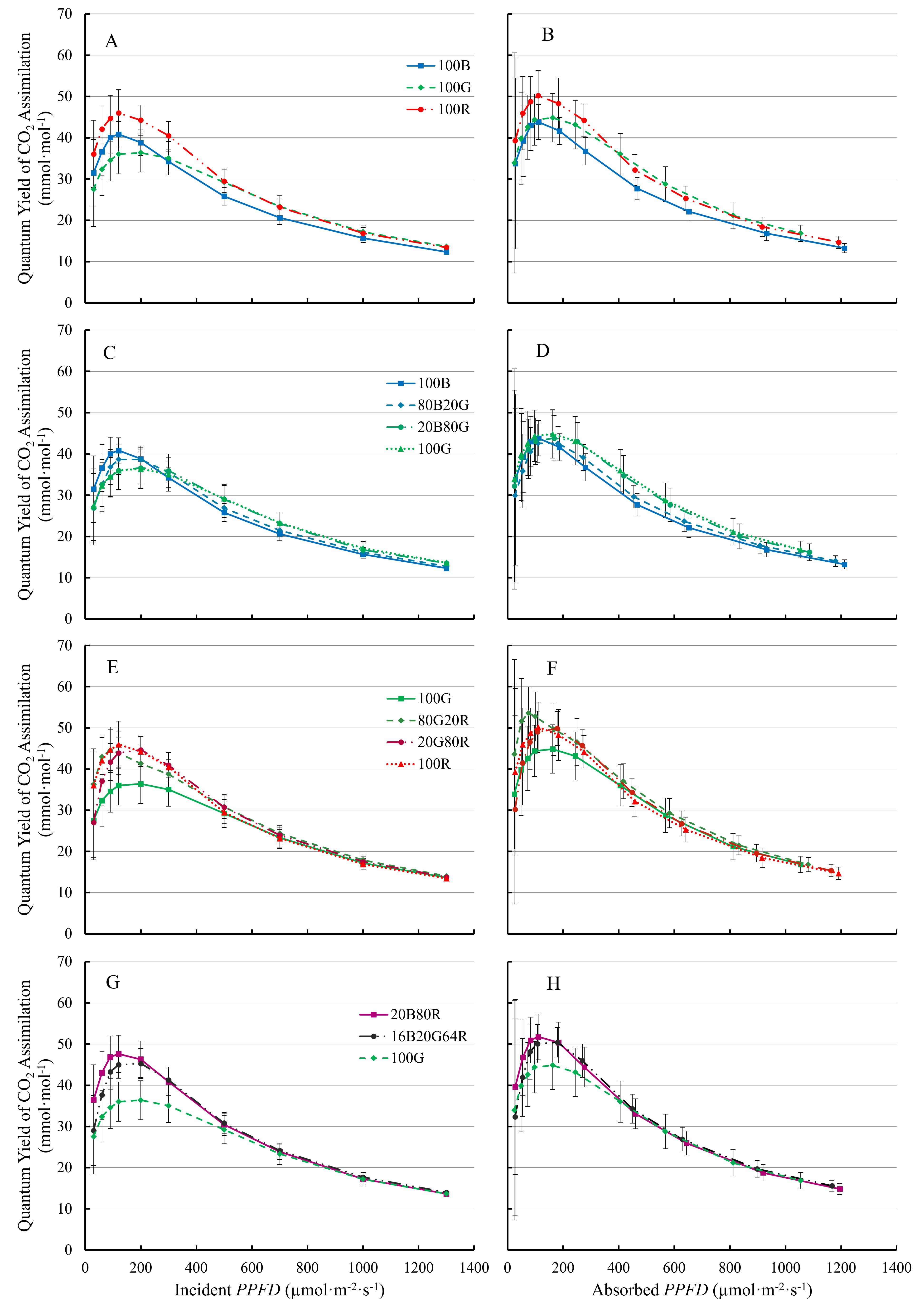

Supplement: Supplementary Figure 1 — (Related to Figure 6) Quantum yield of CO2 assimilation of “Green Towers” lettuce as a function of incident (QYinc) (A,C,E,G) and absorbed PPFD (QYabs) (B,D,F,H) under nine light spectra (see Table 1). Error bars represent standard deviation (n = 9). [file Image_1.TIF]

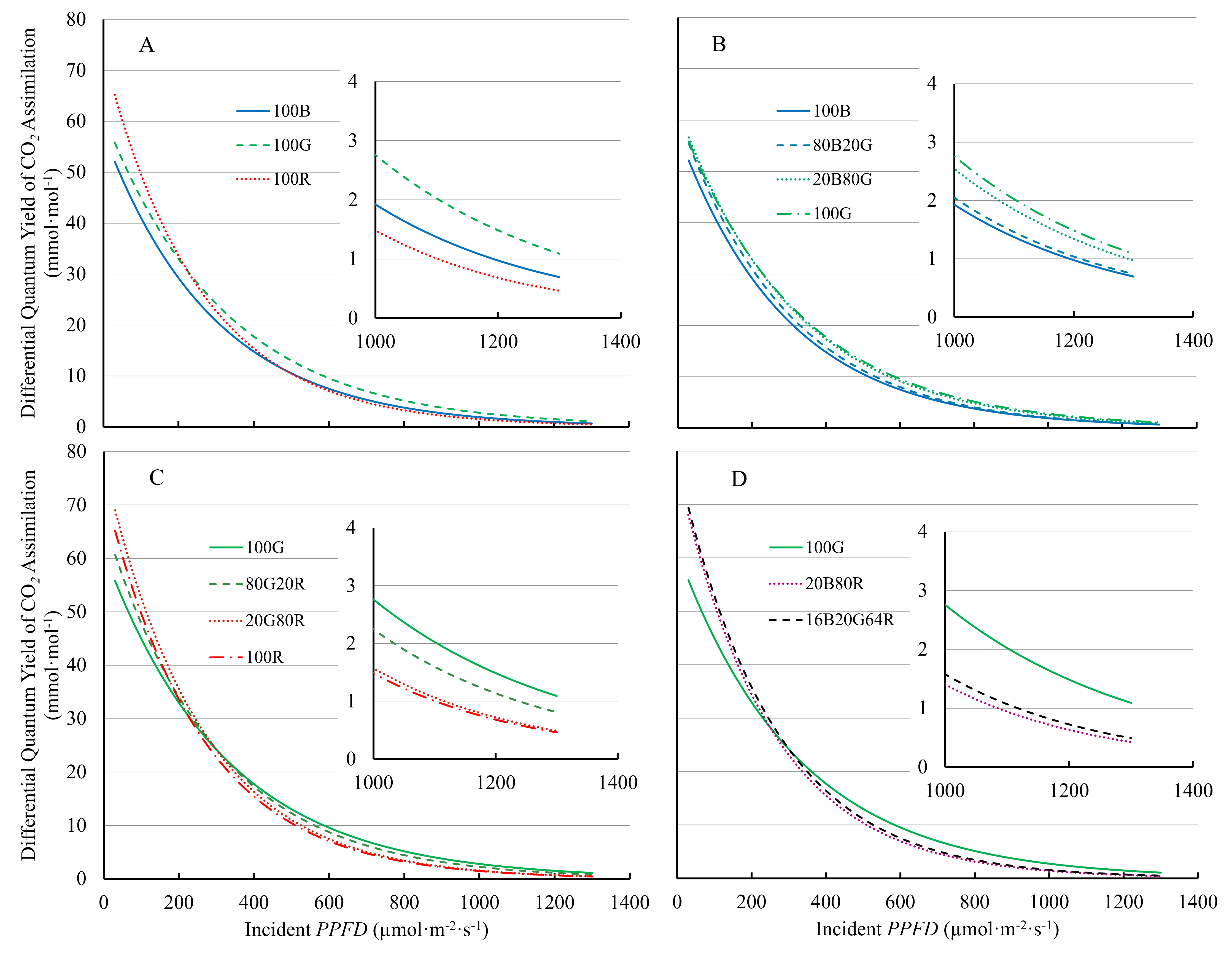

Supplement: Supplementary Figure 2 — (Related to Figure 7) Differential quantum yield of CO2 assimilation (differential QY) of “Green Towers” lettuce under nine light spectra as a function of the PPFD. Inserts show differential QY at PPFDs of 1,000–1,300 μmol⋅m–2 s–1s to better show differences at high PPFD (note the different y-axis scale). The composition of the nine light spectra is shown in Table 1. The light spectra in the graphs are (A) 100B, 100G and 100R; (B) 100B, 80B20G, 20B80G and 100G; (C) 100G, 80G20R, 20G80R and 100R; and (D) 20B80R, 16B20G64R and 100G. [file Image_2.TIF]

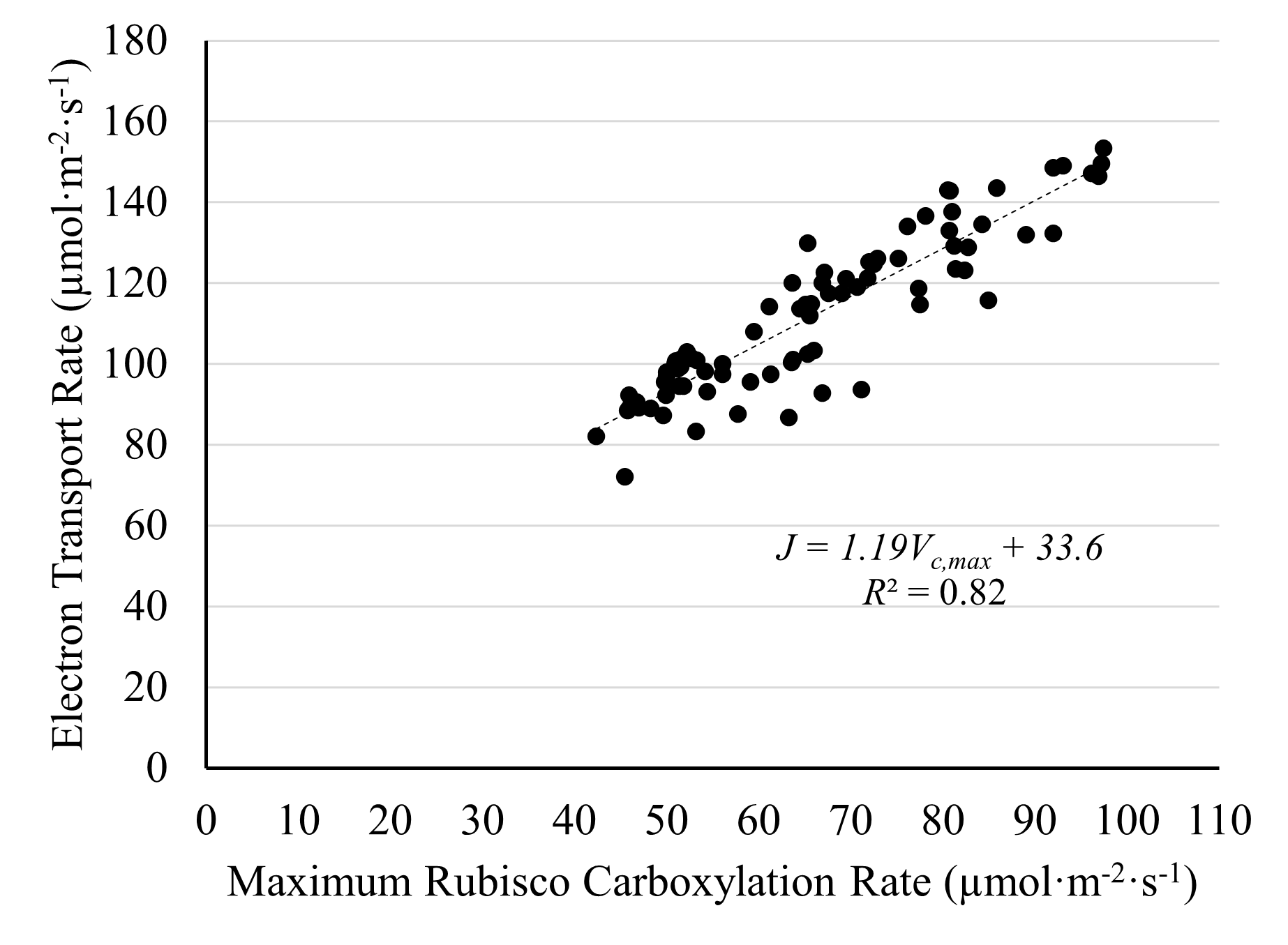

Supplement: Supplementary Figure 3 — (Related to Figure 6) The correlation between electron transport (J) and maximum Rubisco carboxylation rate (Vc,max) of “Green Towers” lettuce estimated from A/Ci curves under PPFD (1000 μmol m–2 s–1) under nine light spectra (p < 0.001). [file Image_3.TIF]

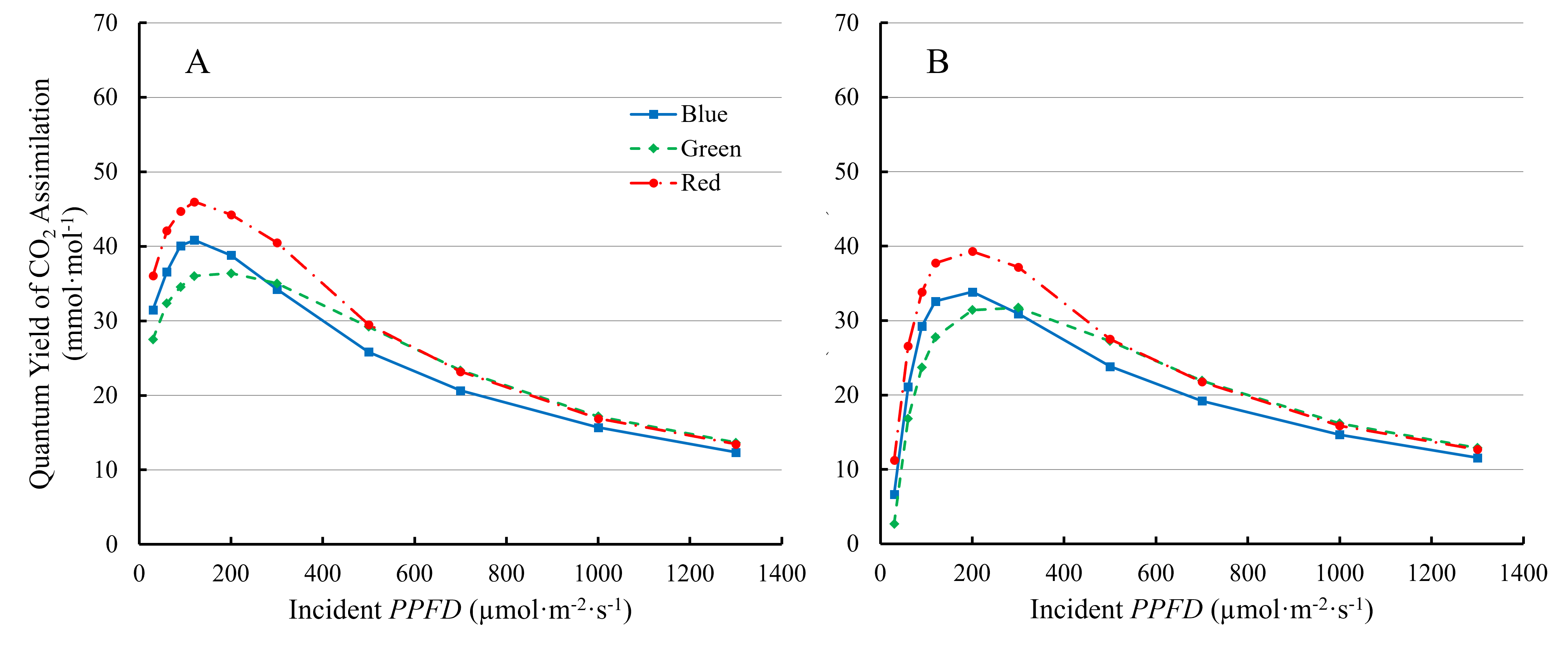

Supplement: Supplementary Figure 4 — (Related to Figure 6) The comparison between QYinc before (A) and after (B) correcting for light-suppression of respiration under blue, green, and red LED light. Note that the initial increase in QYinc became more pronounced after correction of light suppressed respiration. [file Image_4.TIF]

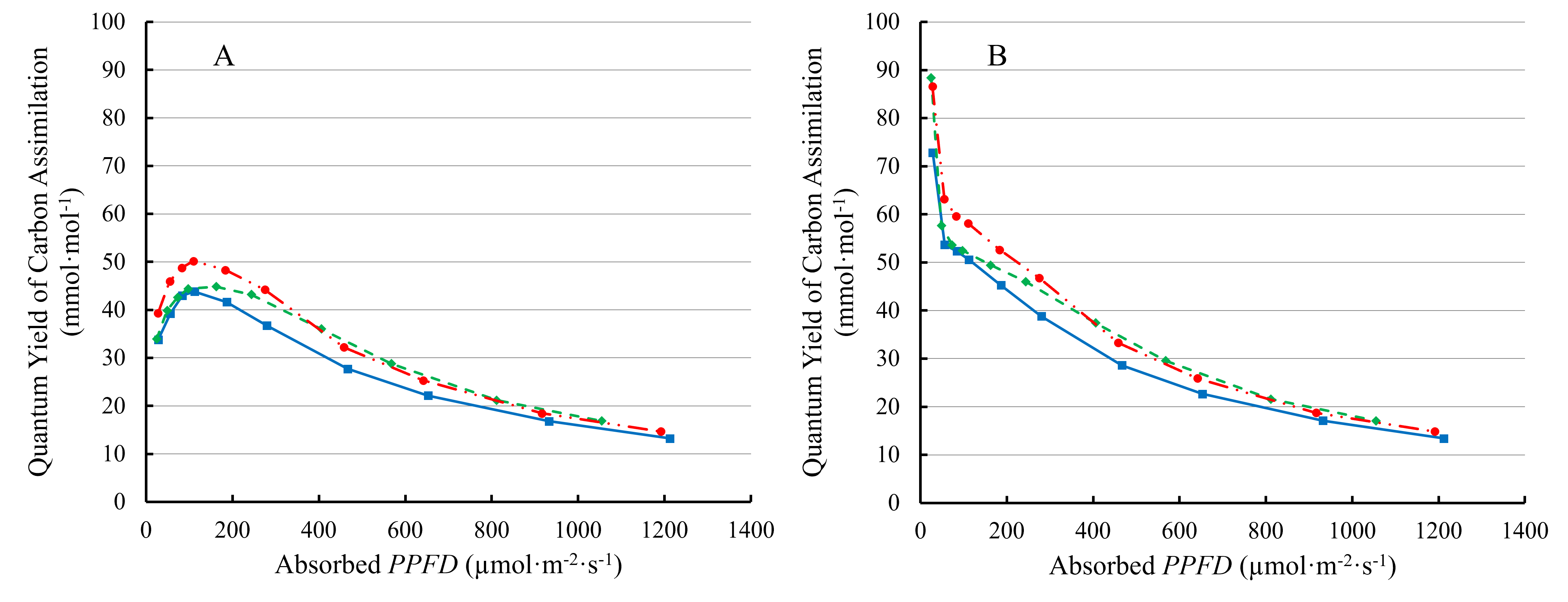

Supplement: Supplementary Figure 5 — The comparison between QYabs before (A) and after (B) correcting for alternative electron sinks under blue, green, and red LED light. Assuming a simplified electron sink that diverts energy of 15 μmol m–2 s–1 of absorbed photons (an arbitrary value used for illustrative purposes only) away from the Calvin cycle under all PPFDs, the corrected QYabs was calculated based on remaining photons available to support Calvin cycle processes (B). Note that the pattern of QYinc after correcting of alternative electron sink (B) is similar to quantum yield of PSII measured by chlorophyll fluorescence by Weaver and van Iersel (2019). [file Image_5.TIF]
