# Supplementary material for: Photosynthetic Physiology of Blue, Green, and Red Light: Light Intensity Effects and Underlying Mechanisms
Source: Front Plant Sci. 2021 Mar 5;12:619987. doi: 10.3389/fpls.2021.619987 (PMC7977723; doi:10.3389/fpls.2021.619987)
Supplement: Supplementary Table 1 — Dark respiration rate (Rd), maximum quantum yield of CO2 assimilation (QYm,inc) and maximum gross assimilation rate (Ag,max) of “Green towers” lettuce derived from the light response curves for nine different spectra using Eq. 1. The light response curves are shown in Figure 3. *See light composition of nine lights presented here in Table 1. [file Table_1.docx]

*Supplementary Table 1. Dark respiration rate (R_d_), maximum quantum yield of CO_2_ assimilation (QY_m,inc_) and maximum gross assimilation rate (A_g,max_ ) of ‘Green towers’ lettuce derived from the light response curves for nine different spectra using equation 1. The light response curves are shown in fig. 3.*

| **Light spectrum^*^** | ***R_d_*** (µmol·m^-2^·s^-1^) | ***QY_m,inc_*** (mol·mol^-1^) | | ***A_g,max_*** (µmol·m^-2^·s^-1^) | |
| --- | --- | --- | --- | --- | --- |
| 100B | 2.30 | 0.058 | c | 17.0 | d |
| 80B20G | 2.73 | 0.061 | c | 18.1 | c |
| 20B80G | 2.96 | 0.062 | c | 19.5 | ab |
| 100G | 2.89 | 0.061 | c | 19.8 | a |
| 80G20R | 2.59 | 0.067 | b | 19.8 | a |
| 20G80R | 3.32 | 0.078 | a | 19.9 | ab |
| 100R | 2.81 | 0.073 | ab | 18.8 | bc |
| 20B80R | 2.88 | 0.077 | a | 19.2 | ab |
| 16B20G64R | 3.21 | 0.078 | a | 20.0 | a |

*^*^See light composition of nine lights presented here in Table 1*
